# An Uncertainty-Based Distributed Fault Detection Mechanism for Wireless Sensor Networks

**DOI:** 10.3390/s140507655

**Published:** 2014-04-25

**Authors:** Yang Yang, Zhipeng Gao, Hang Zhou, Xuesong Qiu

**Affiliations:** State Key Laboratory of Networking and Switching Technology, Beijing University of Posts and Telecommunications, No.10 Xitucheng Road, Haidian District, Beijing 100876, China; E-Mails: gaozhipeng@bupt.edu.cn (Z.G.); zhouhangcauc@163.com (H.Z.); xsqiu@bupt.edu.cn (X.Q.)

**Keywords:** fault detection, uncertainty, evidence fusion, data missing, information entropy

## Abstract

Exchanging too many messages for fault detection will cause not only a degradation of the network quality of service, but also represents a huge burden on the limited energy of sensors. Therefore, we propose an uncertainty-based distributed fault detection through aided judgment of neighbors for wireless sensor networks. The algorithm considers the serious influence of sensing measurement loss and therefore uses Markov decision processes for filling in missing data. Most important of all, fault misjudgments caused by uncertainty conditions are the main drawbacks of traditional distributed fault detection mechanisms. We draw on the experience of evidence fusion rules based on information entropy theory and the degree of disagreement function to increase the accuracy of fault detection. Simulation results demonstrate our algorithm can effectively reduce communication energy overhead due to message exchanges and provide a higher detection accuracy ratio.

## 1. Introduction

Sensors can be rapidly deployed into large areas and perform monitoring tasks by autonomous wireless communication methods. In disaster prevention applications, for example, nodes detect and estimate environmental information, and then forecast when and where a natural calamity may occur. Although users always hope that the network will provide excellent monitoring and gathering functions, it seems inevitable that sensors to experience faults caused by some extrinsic and intrinsic factors. Generally, a fault is an unexpected change in the network, which leads to measurement errors, system breakdown or communication failure.

Faults are generally classified as crash, timing, omission, incorrect computation, fail breakdown, authenticated Byzantine, *etc.* [1]. From another point of view, crash faults are classified as communication faults, since under those conditions, a sensor can't communicate with others because it has a failure in its communication module or the link is down. Contrarily, all other faults are viewed as data faults, which means the faulty sensors can communicate with each other, but the sensed or transmitted data is not correct [2,3]. To avoid erroneous judgments due to faults, broken-down nodes should be detected and isolated from other functioning nodes. Fault detection should make an unambiguous decision about whether the behavior of a sensor deviates from other common measurements.

Sensors always form a local view of the fault state of sensors by collecting measurements from their one-hop neighbors. Neighbor cooperation is one approach to fault detection, whereby a sensor uses neighbor measurements to decide its own fault state collaboratively [4–6]. This is demonstrated to be efficacious for fault information collection and diagnosis because it alleviates the overheads of sink nodes or base stations in order to avoid network bottlenecks. Accordingly, a novel challenge for fault detection in wireless sensor networks (WSNs) is how to reduce the energy consumption when exchanging messages is the main means of fault detection in the distributed environment. Moreover, the dynamic network topology and signal loss caused by long propagation delays and signal fading influence the efficiency of fault detection in some advanced medical care or battlefield response applications.

**Figure 1.**
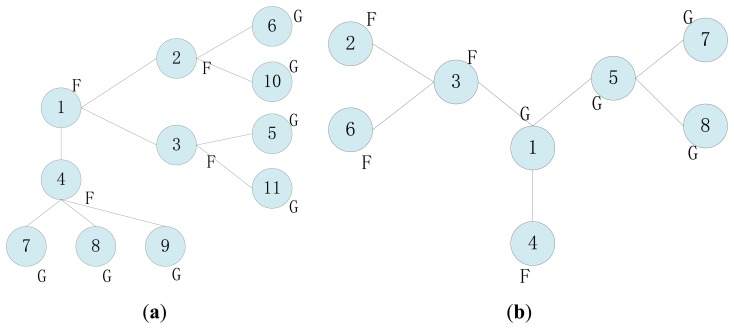
Fault detection illustration.

In the majority voting algorithm based on neighbor cooperation detection, the normal measurements of sensors that are located close to each other are assumed to be spatially correlated, while the fault data are uncorrelated. The tendency state of a sensor is determined as possibly faulty (*LF*) or possibly good (*LG*) by comparing its own readings with those of its one-hop neighbors in a voting process. If the number of *LG* neighbors that have correlated readings is greater than or equal to half, then it is fault-free, otherwise it is deemed faulty. The weighted voting approach uses geographic distance or degree of trust as the deciding factor when calculating the sensor state, but these methods perform in WSNs better based on the hypothesis of higher average connectivity degree. Actually, sensors are usually deployed in a lower connectivity environment, in which exchanged readings are too few to make an appropriate comparison and decision (e.g., in Figure 1a frontier node only has one neighbor). Then the detection accuracy rate decreases as the fault rate increases. Moreover, the faults caused by attacks are unevenly distributed in the case of intrusion monitoring because the hostile signals without a fixed routing will randomly affect or tamper with readings.

In this paper, we mainly focus on sensing faults other than communication faults. After analyzing the defects of traditional algorithms, we present an Uncertainty-based Distributed Fault Detection (uDFD) mechanism for wireless sensor networks. The main contributions of uDFD are as follows:

(1) Propose the uncertainty-based distributed fault detection algorithm, which can avoid decreasing fault detection accuracy when the failure ratio becomes higher. In addition, the accuracy of fault detection remains at a high level regardless of a lower connectivity scene;
(2) Data loss will influence the fault judgment because each sensor determines its own state step by step according to its neighbors' measurements. The paper represents a data forecast model based on a Markov decision processes for filling in lost data to provide reference data for others' state determinations;
(3) We classify two types of sensors' tendency states: Possible Good (*LG*) and Undetermined (*Un*). *LG* nodes contribute to judge nodes' ultimate state. The *Un* nodes are both in an uncertainty status, so we must determine the ultimate status of an *Un* node. Here we design belief probability assignment (*BPA*) functions for different evidences that reflect the states of *Un* nodes. What's more, an evidence fusion rule based on information entropy theory is used to avoid evidence conflicts.

The rest of the paper is organized as follows: Section 2 describes some related works in the area of fault detection in WSNs. Section 3 introduces our Uncertainty-based Distributed Fault Detection algorithm (uDFD) and the concrete mechanisms involved. Section 4 depicts the simulation results with respect to typical fault detection algorithms like DFD and IDFD, and demonstrates our algorithm's efficiency and superiority. In Section 5, we conclude the paper.

## 2. Related Works

In this section, we briefly review related works in the area of distributed and centralized fault detection in WSNs. The authors in [4] proposed and evaluated a localized fault detection scheme (DFD) to identify faulty sensors. An improved DFD scheme was proposed by Jiang in [5]. Neighbors always exchange sensing measurements periodically, therefore a sensor judges its own state (as good or faulty) according to neighbors' values. A faulty identification algorithm reported in [7] is completely localized and requires lower computational overhead, and it can easily be scaled to large sensor networks. In the algorithm, the reading of a sensor is compared with its neighbors' median readings. If the difference is large or large but negative, the sensor is deemed as faulty. If half of neighbors are faulty and the number of neighbors is even, the algorithm cannot detect faults.

Krishnamachari and co-workers proposed in [8] a distributed solution for the canonical task of binary detection of interesting environmental events. They explicitly take into account the possibility of measurement faults and develop a distributed Bayesian scheme for detecting and correcting faults. Each sensor node identifies its own status based on local comparisons of sensed data with some thresholds and dissemination of the test results [9]. Time redundancy is used to tolerate transient sensing and communication faults. To eliminate the delay involved in *z* time redundancy scheme, a sliding window is employed with some data storage for comparison with previous results.

The MANNA scheme [10] creates a manager located externally to the WSN. It has a global vision of the network and can perform complex tasks that would not be possible inside the network. Management activities take place when sensor nodes are collecting and sending temperature data. Every node will check its energy level and send a message to the manager/agent whenever there is a state change. The manager can then obtain the coverage map and energy level of all sensors based upon the collected information. To detect node failures, the manager sends GET operations to retrieve the node state. Without hearing from the nodes, the manager will consult the energy map to check its residual energy. In this way, MANNA architecture is able to locate faulty sensor nodes. However, this approach requires an external manager to perform the centralized diagnosis and the communication between nodes and the manager is too expensive for WSNs.

Tsang-Yi *et al.* [11] proposed a distributed fault-tolerant decision fusion in the presence of sensor faults. The collaborative sensor fault detection (CSFD) scheme is proposed to eliminate unreliable local decisions. In this approach, the local sensors send their decisions sequentially to a fusion center. This scheme establishes an upper bound on the fusion error probability based on a pre-designed fusion rule. This upper bound assumes identical local decision rules and fault-free environments. They proposed a criterion to search the faulty sensor nodes which is based on this error boundary. Once the fusion center identifies the faulty sensor nodes, all corresponding local decisions are removed from the computation of the likelihood ratios that are adopted to make the final decision. This approach considers crash and incorrect computation faults.

In [12], a taxonomy for classification of faults in sensor networks and the first on-line model-based testing technique are introduced. The technique considers the impact of readings of a particular sensor on the consistency of multi-sensor fusion. A sensor is most likely to be faulty if its elimination significantly improves the consistency of the results. A way to distinguish random noise is to run a maximum likelihood or Bayesian approach on the multi-sensor fusion measurements. If the accuracy of final results of multisensory fusion improves after running these procedures, random noise should exist. To get a consistent mapping of the sensed phenomena, different sensors' measurements need to be combined in a model. This cross-validation-based technique can be applied to a broad set of fault models. It is generic and can be applied to an arbitrary system of sensors that use an arbitrary type of data fusion. However, this technique is centralized. Sensor node information must be collected and sent to the base station to conduct the on-line fault detection.

Miao *et al.* [13] presented an online lightweight failure detection scheme named Agnostic Diagnosis (AD). This approach is motivated by the fact that the system metrics of sensors (e.g., radio-on time, number of packets transmitted) usually exhibit certain correlation patterns. This approach collects 22 types of metrics that are classified into four categories: (1) timing metrics (e.g., RadioOnTimeCounter). They denote the accumulative radio-on time; (2) traffic metrics (e.g., TransmitCounter). They record the accumulative number of packets transmitted by a sensor node; (3) task metrics (e.g., TaskExecCounter). This is the accumulative number of tasks executed; (4) other metrics such as Parent Change Counter, which counts the number of parent changes. AD exploits the correlations between the metrics of each sensor using a correlation graph that describes the status of the sensor node. By mining through the periodically updated correlation graphs, abnormal correlations are detected in time. Specifically, in addition to predefined faults (*i.e.*, with known types and symptoms), silent failures caused by Byzantine faults are considered.

Exchanging too many messages for fault detection will cause not only a degradation of the network quality of service, but also a huge burden on the limited energy of sensors. Hence, we design an uncertainty-based distributed fault detection based on neighbor cooperation in WSNs. It adopts auto-correlated test results to describe different sensing states from day to day, and the information entropy-based D-S evidence theory will be introduced to deduce actual states for undetermined nodes.

## 3. Uncertainty-Based Fault Detection Mechanism

### 3.1. The DFD and IDFD Schemes and Their Drawbacks

This section presents the DFD algorithm proposed by Chen [4] and IDFD algorithm described by Jiang [5] to give an overview of distributed fault detection, and then analyzes these algorithms' drawbacks. Chen [4] introduced a localized fault detection method by exchanging measures in WSNs. It is assumed that *x_i_* is the measurement of node *i*. We define $d_{ij}^{t}$ to represent the measured difference between node *i* and *j* at time *t*, while $\Delta d_{ij}^{\Delta t_{l}}$ is measurement difference from time *t_l_* to *t_l_*_+1_:

(1) $$d_{ij}^{t}=x_{i}^{t}-x_{j}^{t}$$

(2) $$\Delta d_{ij}^{\Delta t_{l}}=d_{ij}^{t_{l+1}}-d_{ij}^{t_{l}}=(x_{i}^{t_{l+1}}-x_{j}^{t_{l+1}})-(x_{i}^{t_{l}}-x_{j}^{t_{l}})$$

When $\left|d_{ij}^{t}\right|$ is less than or equal to a predefined threshold *θ*_1_, we will consider a test result *c_ij_* is set to 0, or else it continuously calculates $\left|\Delta d_{ij}^{\Delta t_{l}}\right|$. If $\left|\Delta d_{ij}^{\Delta t_{l}}\right|>\theta_{2}$ (*θ*_2_ is also a predefined threshold), then *c_ij_* = 1, otherwise *c_ij_* = 0. Here the expression *c_ij_* = 1 means node *i* and node *j* are possibly in different states. Next, the tendency status (possibly a faulty *LF* or possibly a good *LG*) is determined according to following formula [14]:

(3) $$T_{i}=\{\begin{array}{cc} LF & if\underset{j\in N_{i}}{\text{∑}}c_{ij}\geq \left\lceil \left|N_{i}\right|/2\right\rceil \\ LG & \text{otherwise} \end{array}$$

where ⌈|*N_i_*|⌉ is the number of one-hop neighbors of node *i*. The formula states that a sensor is deemed to be possibly good only if there are less than ⌈|*N_i_*|/2⌉ neighbors whose test results are 1. In order to process the second round test, each node needs to send its tendency state to its one-hop neighbors. In the DFD algorithm, in the end state the node *Z_i_* is decided to be fault-free only if a difference *γ* is greater than or equal to ⌈|*N_i_*|/2⌉, otherwise *i* is undetermined. Here *γ* = Σ(1 − *c_ij_*) − Σ*c_ij_* = Σ(1 − 2*c_ij_*) (∀*j* ∈*N_i_, T_j_* = *LG*). In order to promote identification efficiency for undetermined sensors, these nodes repeatedly check whether their neighbor's state is fault-free or not. If such a neighbor exists, then the sensor is faulty (fault-free) according to the test result 1(0) between them. A sensor may not decide its own state because the states of neighbors are in conflict, e.g., *Z_j_* = *Z_k_* = *GOOD*. At the same time, *c_ji_* ≠ *c_ki_*. Then *Z_i_* is *GOOD* if *T_i_* = *LG*, or else *Z_i_* is *FAULT*.

Jiang [5] considers the determinant condition Σ*_j_*_∈_*_N_i__*_&_*_T_J__*_=_*_LG_*(1 − 2*c_ij_*) ≥ ⌈|*N_i_*|/2⌉ in the DFD algorithm is too harsh and this will lead some normal nodes to be misdiagnosed as faulty, so the determinant condition for a normal node is amended as:

(4) $${\text{∑}}_{j\in N_{i}\&T_{J}=LG}c_{ij}<{\left\lceil \left|N_{i}\right|/2\right\rceil }_{T_{j}=LG}$$

If there is no tendency status of a neighbor as *LG*, then the final determinant status is set as normal (faulty) based on *T_i_* = *LG* (*T_i_* = *LF*). Although this mechanism promotes the fault detection accuracy to a certain extent through simulation demonstration, it doesn't have a clear way to resolve conflicts or erroneous judgments as illustrated in Figure 1.

In Figure 1a, it calculates *c*_12_ = 0, *c*_13_ = 0, and *c*_14_ = 0 for node 1. Then *T*_1_ is set as *LG* according to Equation (3). In the same way, we get *T*_2_ = *LF, T*_3_ = *LF, T*_4_ = *LF*. Node 1 has no neighbor whose tendency status is *LG*, and then the final determinant status is set as normal based on the rule of *T_i_* = *LG*. This is an obvious erroneous judgment.

The tendency states in Figure 1b are calculated as follows: *T*_1_ = *LF, T*_2_ = *LG, T*_3_ = *LF, T*_4_ = *LG*. For node 1, Σ*_j_*_∈_*_N_i___&__T_J__*_=_*_LG_c_ij_* = 1_|_*_T_*__3_=_*_LG_* + 0_|_*_T_*__5_=_*_LG_* = 1, and ⌈|*N_i_*|/2⌉*_T_j__*_=_*_LG_* = 2/2 = 1. The node 1 is decided as faulty according to Equation (4). Actually, node 1 is a normal sensor. Node 1 will make a mistake when the number of normal neighbors equals the number of faulty neighbors. The premise is that their initial detection tendency states are *LG*.

By analyzing misjudgment conditions of traditional algorithms, a defect is that an indeterminacy occurs on the condition “=” in Equation (4), and thus the node is not reducible to good or faulty. Another is that these algorithms ignore the effect of sensors' own measurements which are approximate at the same time on adjacent days (e.g., 8 June and 9 June). The analogous and historical readings of the same node contribute to determine the faulty state under vague conditions.

Moreover, most distributed fault detection mechanisms assume that sensors have the ability to acquire every measurement and cooperatively judge the state of each other. When the sensor's communication module has a failure, but the acquisition module is active, the readings can't be perceived by the sensor. In a distributed collaborative process, nodes diagnose data faults based primarily on neighbors' data. Once a neighbor's data is missing, it will affect the accuracy of fault diagnosis, e.g., in Figure 1b, node 4 can't determine its own status when node 1 has no data.

### 3.2. Uncertainty-Based Distributed Fault Detection Algorithm

In the paper, we mainly resolve the following problems: (1) data missing before exchanging readings; (2) misjudgments caused by indeterminacy conditions. The problem of missing data due to communication faults will affect the determination accuracy when comparing neighbors' measurements. To solve the data loss, a faulty sensing node should fill in the missing measurements to provide the reference. Secondly, the represented algorithm adopts the auto-correlated test results to describe the status of differences between different days. Finally, those undetermined appearances may occur in the above-mentioned section. The information entropy and the degree of disagreement function combined in evidence fusion theory are improved accordingly to help to deduce their actual states. In addition, using information entropy in the evidence fusion can reduce evidence conflicts and increase detection accuracy.

#### 3.2.1. Definitions

We list the notations in the uDFD algorithm as follows:

- *p*: Probability of fault of a sensor;
- *N_i_*: A set of neighbors of node *i*;
- $x_{i}^{D,t}$: Measurement value of node *i* at time *t* on day *D*;
- ⌈|*N_i_*|⌉: Number of one-hop neighbors of node *i*;
- $d_{ij}^{t}$: Measurement difference between node *i* and *j* at time *t* on the same day according to Formula (1);
- $\Delta d_{ij}^{\Delta t_{l}}$: Measurement difference between node *i* and *j* from time t*_l_* to t*_l+_*_1_ on the same day according to Formula (2);
- $\Delta d_{ii}^{D,t}$: Measurement difference of node *i* at the same time *t* on different day;
- *c_ij_*: Test result between node *i* and *j, c_ij_* ∈ {0, 1};
- *T_i_*: Tendency value of a sensor, *T_i_* ∈ {*LG, Un*};
- *Z_i_*: Determined detection status of a sensor, *Z_i_* ∈ {GOOD,FAULT};
- *θ_1_*, *θ_2_*, *θ_3_*: Predefined threshold values about $d_{ij}^{t}$, $\Delta d_{ij}^{\Delta t_{l}}$, $\Delta d_{ii}^{D,t}$;
- *Num_i_*({*G*}): Number of good neighbors of node *i*;
- *Num_i_*({*F*}): Number of faulty neighbors of node *i*.

#### 3.2.2. Fault Detection

The main processes of the uDFD algorithm based on neighbor cooperation are summarized as follows. The key technology for solving the two problems is described in Sections 3.2.3 and 3.2.4.

Stage 1: Each sensor acquires the readings from its own sensing module. If no data is acquired, then it fills up the missing data. After that, it exchanges the measurement at time *t* on day *D* with its neighbors and calculates the test result *C_ij_* (It's assumed that *C_ij_* = 0 at the initial time):
1:**If**
$\left|\Delta d_{ii}^{D,t}\right|\leq \theta_{3}$, **then** set *C_ij_* = 0;2:**else**
*C_ij_* = 1;3:**end if**4:**If**
$\left|d_{ij}^{t}\right|>\theta_{1}$, **then**
*C_ij_* = 15:**else if**
$\left|d_{ij}^{t}\right|\leq \theta_{1}\&\&\left|\Delta d_{ij}^{\Delta t_{l}}\right|>\theta_{2}$, **then**
*C_ij_* = 1;6:**else**
*C_ij_* = 0;7:**end if**8:Repeat the above steps until all of test results about neighbors are obtained.
Stage 2: Node *i* generates the tendency value based on *c_ij_* (∀*j*):
9:**If**
$\underset{j}{\text{∑}}c_{ij}+c_{ii}<\frac{\left\lceil \left|N_{i}\right|\right\rceil +1}{2}$, **then**
*T_i_* = *LG*;10:**else**
*T_i_* = *Un*;11:**end if**12:Broadcast the tendency status if *T_i_* = *LG*.
Stage 3: Calculate the determined status of *LG* nodes:
13:**If**
*T_i_* = LG && (∃*j*)*j* ∈ {*LG*};14:**If**
$\underset{j=LG}{\text{∑}}C_{ij}<{\frac{Num(N_{s_{i}})+1}{2}}_{j=LG}$, **then**
*Z_i_* = Good;15:**else**
*Z_i_* = Fault;16:**end if**17:**else if**
*T_i_* = *LG* && no any neighbor is *LG*, **then**
*T_i_* = *Un*;18:**end if**19:A *LG* node can determine its own status (good or faulty), and only good sensors broadcast their states in order to save transmission overheads.
Stage 4: A node whose tendency status is *Un* determines the actual state by using entropy-based evidence combination mechanism:
20:Node *i* (*i* ∈ {*LE,Un*}) receives the evidence of good neighbors.21:Combine the evidences generated by measurements by adopting information entropy-based evidence fusion, and acquire the combined *BPA* functions m*({*G*}), m*({*F*}), and m*(Ψ);22:Node *i* finds the node *j* which matches the min⌈*m_j_*({*G*} − m*({*G*}))⌉;23:**if**
*c_ij_* = 1, **then**
*Z_i_* = FAULT, **else**
*Z_i_* = GOOD;24:**end if**25:Determined node broadcasts its status if it's a good sensor.

Broadcasting not only uses up nodes' energy but also occupies the channel bandwidth, so the main method of saving energy consumption in our algorithm is that only particular states in different stages (*LG* and *GOOD*) are broadcast. In Step 12, only the node whose tendency status is equal to *LG* broadcasts the value. The reason is that only *LG* neighbors participate in final state determination in Step 14. Similarly, only good sensors broadcast their states in order to save energy transmission overhead.

#### 3.2.3. Missing Data Preprocessing Mechanism

In the paper, we mainly focus on sensing faults rather than communication faults. When missing data occurs because of a sensing fault, it will affect the accuracy of fault diagnosis. This means $X_{i}^{D,t}$ has been lost because the communication module has failed, which subsequently influences the reference data for other sensors' faulty state determination. It is necessary for node *i* to fill in the missing data and send it to neighbors. In this section, we use a Markov decision processes based on neighbors' historical data to predict the current missing measurement values of node *i*. Relying the features of Markov theory which can reflect the influence of random factors and extension to the stochastic process which is dynamic and fluctuating is considered and we combine the historical data of node *i* with its neighbors' historical data, and then form a fusion historical data vector, which can be adaptively adjusted according to the significance of neighbors' measurements. Therefore, the state transition matrix of Markov is adopted to predict the value and sign of the reading difference between two days. The steps for data missing preprocess preprocessing are as follows:

Steps:

1. For each node *j* ∈ *N_i_*, where *N_i_* is the set of all the neighbors of node *i*, fetch the previous *m* historical measurements of node *j*, and these historical measurements correspond to an *m* dimensional vector ***V****_j_*, that is $V_{j}=(X_{j}^{D-m,t},X_{j}^{D-m+1,t},...,X_{j}^{D-1,t})$;
2. Calculate the reputation value *C_ij_* for each neighbor of node *i*, that is for each node *j* ∈ *N_i_*, we have *C_ij_* = *e*^−^*^λ_i_^*, where $\lambda_{i}=\frac{1}{m}{\text{∑}}_{k=1}^{m}\left|X_{j}^{D-k,t}-X_{i}^{D-k,t}\right|$. Note that for a different node *i*, node *j* has different reputation values and a smaller value for *λ_i_* will increase the reputation value of node *j*;
3. Here we introduce Mahalanobis distance to evaluate the similarity distance between node *i* and its neighbors. Then the prediction results should keep Mahalanobis distance changes within a predefined threshold. For each node *j* ∈ *N_i_*, calculate the Mahalanobis's distance *D*(***V****_i_*,***V****_j_*) between vectors ***V****_i_* and ***V****_j_*, in order to evaluate the similarity of node *i* and all its neighbors. That is $D(V_{i},V_{j})=\sqrt{{\left(V_{i}-V_{j}\right)}^{T}{\text{∑}}^{-1}(V_{i}-V_{j})}$, where Σ is the covariance matrix of ***V****_i_* and ***V****_j_*;
4. Assume that $V_{i}^{*}$ is a fusion of the historical measurements of node *i* and all its neighbors, which is used in the Markov decision processes to predict the current measurement of node *i*. It is also an *m* dimensional vector and can be calculated as follows:
  (5) $${\text{V}}_{\text{i}}^{*}=\alpha \times {\text{V}}_{\text{i}}+\beta \times {\text{∑}}_{j=1}^{\left|N_{i}\right|}\left(\frac{C_{ij}}{{\text{∑}}_{k=1}^{\left|N_{i}\right|}C_{ik}}\times {\text{V}}_{\text{j}}\right)$$
  In this data-fusion formula, the historical measurement vector ***V****_j_* is weighted by the reputation value of node *j*, and the factors *α* and *β* (*α* + *β* = 1) indicate to what extent a node trusts itself and neighbors. Here *α* = *β* = 0.5.
5. According to the result of fusion in Step 4, use Markov decision processes to predict the current measurement of node *i*, then we can get $X_{i}^{D,t}$;
6. For each node *j* ∈ *N_i_*, recalculate the Mahalanobis's distance $D^{\prime }(V_{i}^{\prime },V_{j}^{\prime })$ between vectors $V_{i}^{\prime }$ and $V_{j}^{\prime }$. That is $D^{\prime }(V_{i}^{\prime },V_{j}^{\prime })=\sqrt{{\left(V_{i}^{\prime }-V_{j}^{\prime }\right)}^{T}{\text{Σ}}^{\prime -1}(V_{i}^{\prime }-V_{j}^{\prime })}$, here $V_{i}^{\prime }$ and $V_{j}^{\prime }$ are (m + 1) dimensional vectors, and $V_{i}^{\prime }=(X_{i}^{D-m,t},X_{i}^{D-m+1,t},\cdots ,X_{i}^{D-1,t},X_{i}^{D,t})$, $V_{j}^{\prime }=(X_{j}^{D-m,t},X_{j}^{D-m+1,t},\cdots ,X_{j}^{D-1,t},X_{j}^{D,t})$, Σ′ is the covariance matrix of $V_{i}^{\prime }$ and $V_{j}^{\prime }$;
7. If ∀*j* ∈ *N_i_*, $D^{\prime }(V_{i}^{\prime },V_{j}^{\prime })-D\left({\text{V}}_{i},{\text{V}}_{j}\right)\leq \theta$, where *θ* is a predefined threshold, then there is no need to adjust the fusion factor *α* and the predicted value $X_{i}^{D,t}$ can be adopted. Otherwise, the predicted value $X_{i}^{D,t}$ increases the differences between node *i* and node *j*, so the fusion factor *α* needs to be reduced appropriately, in order to decrease the proportion of neighbors' measurements in the calculation of $V_{i}^{*}$;
8. If the fusion factor *α* has been adjusted in Step 7, then return to Step 4. Otherwise, this algorithm ends.

In order to predict the missing data $X_{i}^{D,t}$ in Step 5 of the above algorithm, we draw on the experience of Markov decision processes [15,16]. Firstly, according to $V_{i}^{*}$ which is calculated in Step 4 of the above algorithm, we can get the corresponding vector ${\text{X}}_{\text{i}}=(\left|X_{i}^{\Delta D-m,t}\right|,\left|X_{i}^{\Delta D-m+1,t}\right|,...,\left|X_{i}^{\Delta D-2,t}\right|)$ and it is an (*m* − 1) dimensional vector and can be considered as an independent and identical distributed Markov chain.

Then, we classify the state of each component in vector *X_i_* by an average-standard deviation classification method. Assume that state *s* can be expressed as *E_s_* ∈ [*min_s_, max_s_*], where *min_s_* and *max_s_* indicate the lower bound and upper bound of state *s*.

Then the sample average is:

(6) $$\mu =\frac{1}{m-1}\overset{m}{\underset{j=2}{\text{∑}}}\left|X_{i}^{\Delta D-j,t}\right|$$

The standard deviation is:

(7) $$S=\sqrt{\frac{1}{m-2}\overset{m}{\underset{j=2}{\text{∑}}}{\left(\left|X_{i}^{\Delta D-j,t}\right|-\mu \right)}^{2}}$$

According to central-limit theorem [17], we divide the sliding interval of historical fault data into five states, that is *E*_1_ = (*μ* − 3*S, μ* − *S*), *E*_2_ = [*μ* − *S, μ* − 0.5*S*), *E*_3_ = [*μ* − 0.5*S, μ* + 0.5*S*), *E*_4_ = [*μ* + 0.5*S, μ* + *S*), and *E*_5_ = (*μ* + *S, μ* + 3*S*). The state of each component in the difference vector *X_i_* depending on which sliding interval it belongs to.

The transition probability matrix *P*^(1)^ can be calculated as follows. Assume that $M_{st}^{\left(1\right)}$ indicates the sample numbers that state *E_S_* transfers to state *E_t_* in one step, and *M_S_* indicates the sample numbers of state *E_S_* before transfer. Then we get $p_{st}^{\left(1\right)}=\frac{M_{st}^{\left(1\right)}}{M_{s}}$, where $p_{st}^{\left(1\right)}$ means the transition probability of shifting from state *E_S_* to state *E_t_* by one step. Therefore the 5 × 5 transition probability matrix is:

$$P^{\left(1\right)}=\left(\begin{array}{ccc} p_{11}^{\left(1\right)} & \cdots & p_{15}^{\left(1\right)}\\ \vdots & \ddots & \vdots \\ p_{51}^{\left(1\right)} & \cdots & p_{55}^{\left(1\right)} \end{array}\right)$$

For any component $\left|X_{i}^{\left(\Delta D-j,t\right)}\right|$ (*j* = 1, 2, … *m*), the probability distribution vector is:

(8) $$\pi (\text{D}-\text{j})=(\pi_{1}\left(D-j\right),\pi_{2}\left(D-j\right),\pi_{3}\left(D-j\right),\pi_{4}\left(D-j\right),\pi_{5}\left(D-j\right))$$

Assume that $\left|X_{i}^{\left(\Delta D-2,t\right)}\right|$ is in state *E_3_*, then the probability distribution vector of it is ***π***(***D*** − 2) = (0,0,1,0,0). As the probability distribution vector ***π***(***D*** − 2) and the transition probability matrix *P*^(1)^ are known, then the probability distribution vector of $\left|X_{i}^{\left(\Delta D-1,t\right)}\right|$ is ***π***(***D*** − 1) = ***π***(***D*** − 2) × *P*^(1)^, the corresponding state in *max*{***π****_s_*(***D*** − 1), *s* ∈ {1, 2, 3,4, 5}} is the state $\left|X_{i}^{\left(\Delta D-1,t\right)}\right|$ belongs to. If $\left|X_{i}^{\left(\Delta D-1,t\right)}\right|$ is in state *s*, then the specific value of $\left|X_{i}^{\left(\Delta D-1,t\right)}\right|$ is determined as follows:

(9) $$\begin{array}{ll} \left|X_{i}^{\Delta D-1,t}\right| & ={\text{min}}_{s}\times \frac{\pi_{\text{s}-1}\left(\text{D}-1\right)}{\pi_{\text{s}-1}\left(\text{D}-1\right)+\pi_{\text{s}}\left(\text{D}-1\right)+\pi_{\text{s}+1}\left(\text{D}-1\right)}\\ & +\frac{{\text{min}}_{s}+{\text{max}}_{s}}{2}\times \frac{\pi_{\text{s}}\left(\text{D}-1\right)}{\pi_{\text{s}-1}\left(\text{D}-1\right)+\pi_{\text{s}}\left(\text{D}-1\right)+\pi_{\text{s}+1}\left(\text{D}-1\right)}\\ & +{\text{max}}_{s}\times \frac{\pi_{\text{s}+1}\left(\text{D}-1\right)}{\pi_{\text{s}-1}\left(\text{D}-1\right)+\pi_{\text{s}}\left(\text{D}-1\right)+\pi_{\text{s}+1}\left(\text{D}-1\right)} \end{array}$$

Continue to introduce Markov decision processes to predict the signs (positive and negative) of $\left|X_{i}^{\left(\Delta D-1,t\right)}\right|$. For the vector $X_{i}^{\prime }=(X_{i}^{\left(\Delta D-m,t\right)},X_{i}^{\left(\Delta D-m+1,t\right)},\ldots ,X_{i}^{\Delta D-2,t})$, we define that state *E_1_* corresponds to positive, and state *E_2_* corresponds to negative. Then we get the transition probability $p_{st}^{\prime (1)}=\frac{M_{st}^{\prime (1)}}{M_{st}^{\prime }}$ and the transition probability matrix $P^{\prime (1)}=\left(\begin{array}{cc} p_{11}^{\prime (1)} & p_{12}^{\prime (1)}\\ p_{21}^{\prime (1)} & p_{22}^{\prime (1)} \end{array}\right)$ which reflects the probability of transferences between positive and negative. Also for any component $\left|X_{i}^{\left(\Delta D-j,t\right)}\right|$, *(j* = 1, 2,…,*m)*, the probability distribution vector is $\pi^{\prime }\left(\text{D}-\text{j}\right)=(\pi_{1}^{\prime }\left(D-j\right),\pi_{2}^{\prime }\left(D-j\right)$. Assume that $\left|X_{i}^{\left(\Delta D-2,t\right)}\right|$ is a positive, then the probability distribution vector of it is ***π****′*(***D*** − 2) = (1, 0). As the probability distribution vector ***π****′*(***D*** − 2) and the transition probability matrix *P′*^(1)^ are known, then the probability distribution vector of the sign of $\left|X_{i}^{\left(\Delta D-1,t\right)}\right|$ is ***π****′*(***D*** − 1) = ***π****′*(***D*** − 2) × *P′*^(1)^, the corresponding state in $max\{\pi_{s}^{\prime }(D-1),s\in \{1,2\}\}$ indicates the sign of $\left|X_{i}^{\left(\Delta D-1,t\right)}\right|$.

#### 3.2.4. Information Entropy Based Evidence Confusion

As the *Un* nodes are both in uncertainty status, we need to find a mechanism to determine the status of these nodes. Dempster-Shafer evidence theory is an effective method for dealing with uncertainty problems, but the results obtained are counterintuitive when the evidences conflict highly with each other [18,19].

In the improved evidence fusion algorithm we propose, the possible events can be depicted as evidences. Through combination rules, evidences are aggregated into a comprehensive belief probability assignment under uncertainty conditions. It's assumed that a set of hypotheses about node status is denoted as frame of discernment Θ = {*G, F*}. The symbol *G* represents a good sensor, and *F* is faulty. The power set 2^Θ^ includes all of subsets of Θ. Here 2^Θ^ = {{Φ}, {*G*}, {*F*}, {Ψ}}, each symbol of which respectively represents the hypotheses about *impossible, good, fault*y, and *uncertainty*.

The belief probability assignment (*BPA*) functions of node *i* are depicted as follows:

(10) $$\text{m}:2^{\theta }\to [0,1]$$

(11) $$m_{i}\left(\Phi \right)=0$$

We define the *BPA* function for good status is:

(12) $$m(\{G\})=\{\begin{array}{cc} -\frac{0.5}{\mu_{1}}\xi_{X}+1 & 0\leq \xi_{X}<u_{1}-\sigma_{1}\\ \frac{1-e^{-\frac{{\left(\xi_{X}-u_{1}\right)}^{2}}{2\sigma_{1}^{2}}}}{2} & u_{1}-\sigma_{1}\leq \xi_{X}\leq u_{1}+\sigma_{1}\\ 0.5-\frac{\xi_{X}}{4\mu_{1}} & \xi_{X}>u_{1}+\sigma_{1}\\ 0 & \xi_{X}\geq 2u_{1} \end{array}$$

Similarly, the *BPA* function for faulty status is:

(13) $$m(\{F\})=\{\begin{array}{cc} \frac{\xi_{X}}{4\mu_{1}} & 0\leq \xi_{X}<u_{1}-\sigma_{1}\\ \frac{1-e^{-\frac{{\left(\xi_{X}-u_{1}\right)}^{2}}{2\sigma_{1}^{2}}}}{2} & u_{1}-\sigma_{1}\leq \xi_{X}\leq u_{1}+\sigma_{1}\\ \frac{0.5}{\mu_{1}}\cdot \xi_{X} & \xi_{X}>u_{1}+\sigma_{1}\\ 1 & \xi_{X}\geq 2u_{1} \end{array}$$

The *BPA* function for uncertainty status is:

(14) $$m(\{\Psi \})=\{\begin{array}{cc} \frac{\xi_{X}}{4\mu_{1}} & \xi_{X}<u_{1}-\sigma_{1}\\ e^{-\frac{{\left(\xi_{X}-u_{1}\right)}^{2}}{2\sigma_{1}^{2}}} & u_{1}-\sigma_{1}\leq \xi_{X}\leq u_{1}+\sigma_{1}\\ 0.5-\frac{\xi_{X}}{4\mu_{1}} & \xi_{X}>u_{1}+\sigma_{1}\\ 0 & \xi_{X}\geq 2u_{1} \end{array}$$

Here we design an expectation deviation function *ξ_X_*. It's assumed that the measurement value of nodes at time *t* on day *D* is a random variable, which has the expectation *EX* and variance *σ*^2^. Define $\xi_{X}=\frac{\left|X-EX\right|}{\sigma }$ that means the multiple relation between *σ_X_* and the difference between *X* and *EX*. *ξ_X_i__* indicates the data offset between node *i* and the average of good neighbors. The larger *ξ_X_i__* is, the more probable that the node is faulty. With the increase of *ξ_X_i__, m*({*G*}) reduces, on the contrary, *m*({*F*}) rises.

**Figure 2.**
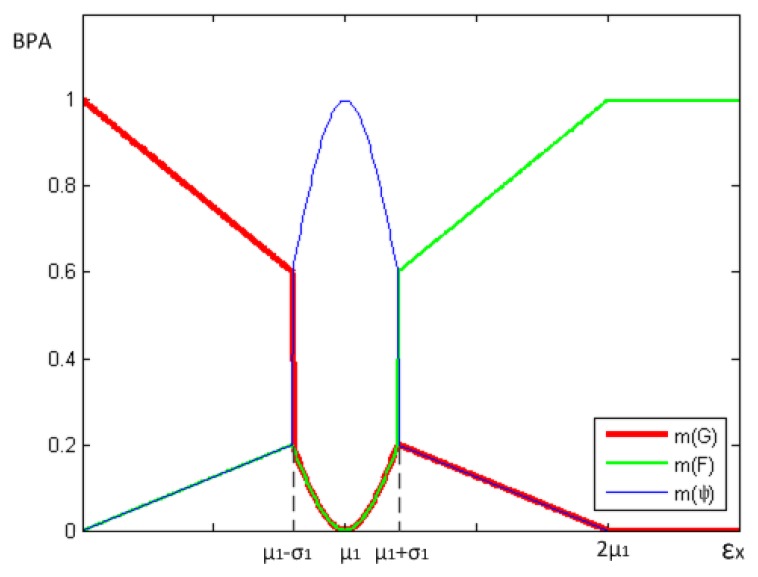
The BPA functions.

In Section 3.1, we have discussed that one of the defects of traditional algorithms is that an indeterminacy occurs for the “=” condition in Equation (4), and thus the node is not reducible to good or faulty. Therefore, we define the range (*μ*_1_ − *σ*_1_, *μ*_1_ + *σ*_1_,) within which the status of this node has higher uncertainty (the probability of this node being fault is moderate) and *m*(Ψ) ∼ *N*(*μ*_1_,*σ*_1_) when *ξ_X_i__* ∈ (*μ*_1_ − *σ*_1_, *μ*_1_ + *σ*_1_). When *ξ_X_i__* = *μ*_1_, *m*({Ψ}) = 1, which means the uncertainty reaches the top (depicted in the Figure 2).The definitions of *m*(*G*), *m*(*F*) and *m*(Ψ) express this meaning above and provide a good description of the influence of changing *ξ_X_i__* on evidence.

In Equation (14), when *ξ_X_i__* ∈ (*μ*_1_ − *σ*_1_, *μ*_1_ + *σ*_1_), we can see that:

(15) $$P(\xi_{x}\geq \mu_{1}+\sigma_{1})\leq e^{-1/2}$$

According to the Chebyshev inequality:

(16) $$\text{p}\{\left|X-EX\right|\geq ɛ\}\leq \sigma^{2}/{ɛ}^{2}$$

Make ε^2^ = *e*^1/2^σ^2^ then ε = *e*^1/4^σ. The formula expands as follows:

(17) $$P\{\frac{\left|X-EX\right|}{\sigma }\geq e^{-1/4}\}\leq e^{-1/2}$$

According to Equations (15) and (16), we get *μ*_1_ + *σ*_1_ = *e*^−1/4^. Here, we define *σ_1_* = 0.1, and then *μ_1_* = 0.68. After all above, *m*({*G*}), *m*({*F*}) and *m*(Ψ) can be calculated by Equations (12)–(14), respectively.

In D-S evidence theory, if there are more than two BPAs that need to be combined, then the combination rule is defined as follows:

(18) $$m\left(Z\right)=\frac{{\text{∑}}_{\cap_{i=1}^{N}B_{i}=Z}\prod_{i=1}^{N}m_{i}(B_{i})}{1-K}$$

where *K* is the mass that is assigned to the empty set Φ, and $K={\text{∑}}_{\cap_{i=1}^{N}B_{i}=\Phi }\prod_{i=1}^{N}m_{i}(B_{i})$. But the traditional Dempster-Shafer evidence has a very obvious disadvantage when being used in our algorithm of fault detection. For example:

$$\begin{array}{c} \begin{array}{cc} m1:m(G)=0.8,m(F)=0.2, & m(\Psi )=0, \end{array}\\ \begin{array}{cc} m2:m(G)=0.8,m(F)=0.2, & m(\Psi )=0. \end{array} \end{array}$$

The fused result is *m*(*G*) = 0.94, *m*(*F*) = 0.06, *m*(Ψ)= 0. However, the result extremely negates *F* in the traditional Dempster-Shafer evidence fusion. Obliterating conflict roughly and running normalization processes leads to extreme differences between *G* and *F.* This will cause errors in the judgment of sensors' states when using uDFD. That is because the node *i* will find the node *j* which matches the min⌈*m_j_*({*G*} − m*({*G*}))⌉. Too extreme evidence will influence the effect of comprehensive evidence. Based on this, we propose a new evidence fusion rule combined with information entropy theory. According to conflicts to the entirety presented by information divergences, we classify evidences into several sets. By fusing the results from different sets, this prevents extreme extension of differences between *G* and *F*. By this evidence fusion algorithm, we can finally determine the nodes' status.

In classical theories of information, Shannon Entropy measures the amount of information, while the amount of information reflects the uncertainty in random events. Considering different evidences should be assigned different fusion weight according to its amount of information, so, in this section, the theories of entropy and the degree of disagreement function which measures the information discrepancy are introduced into combination rules for evidence conflicts and increase the accuracy of fault determination for *Un* nodes. Firstly, we introduce some definitions. The information divergence *D*(*p*∥*q*) between discrete random variables *p* and *q* is defined as below [20]:

(19) $$D(p∥q)=\underset{x}{\text{∑}}p(x)log\frac{p(x)}{q(x)}$$

It is obvious that *D*(*p*∥*q*) ≥ 0 assume that *M_l_* indicates the lth evidence and *M_l_* = (*m*_1_*_l_*, *m*_2_*_l_*,…,*m_nl_*), where *m_il_* is called a focal element. Here, ${\text{∑}}_{i=1}^{n}m_{il}=1,m_{il}\geq 0,0\leq i\leq n,1\leq l\leq s$, where *n* is the number of focal elements in each evidence and *s* indicates the amount of evidences.

*D_l_* is defined as follows:

(20) $$D_{l}=\frac{1}{s}\overset{s}{\underset{j=1}{\text{∑}}}D(M_{l}∥M_{j})=\frac{1}{s}\overset{s}{\underset{j=1}{\text{∑}}}\overset{n}{\underset{i=1}{\text{∑}}}m_{il}\ln\frac{m_{il}}{m_{ij}}$$

It indicates the degree of differences between *M_l_* and the whole evidences. It is determined by the average of the information divergence between *M_l_* and each evidence. After this, define *δ_l_* as the percentage of the whole difference degree that *M_l_* occupies. It is calculated as follows:

(21) $$\delta_{l}=D_{l}/\overset{s}{\underset{i=1}{\text{∑}}}D_{i}$$

According to *δ_l_*, evidences are going to be classified into several subsets. Evidences which have similar *δ_l_* are aggregated in the same subset. Before classification, the demarcation point Δ is confirmed as below, which means the average differences between *δ_l_*:

(22) $$\Delta =\frac{2}{s(s-1)}\overset{s}{\underset{l=1}{\text{∑}}}\overset{s}{\underset{i=l+1}{\text{∑}}}\left|\delta_{l}-\delta_{i}\right|$$

Assume that *C_r_* is the resultant subset and *P* is the collection of *δ_l_*. The pseudo code of the classification algorithm is as follows:
1: *r* = 0;2: While *P* is not empty, do3: Randomly, choose any one element from *P* and put it into *C_r_*. Remark this element as *C_r1_*;4: Remove *C_r1_* from *P*;5: Loop1, for *l* = 1 to s6: Loop2, for *j* = 1 to |*C_r_*| (|*C_r_*| is the cardinality of |*C_r_*|)7: If |*δ_r_* − *C_rj_*| >Δ, then continue loop1;8: End if;9: End loop2;10: Put *δ_l_* into *C_r_* and remove it from *P*;11: End loop1;12: *r*++;13: End while.

After classification, the difference between *δ_l_* of each evidence in the same subset is less than Δ, that is, evidences have a smaller extent of conflict in each subset. Assume that there are *m* subsets and the weight of each subset is defined as *CW_r_* = |*C_r_*|/*s*, where |*C_r_*| is the number of evidences in each subset. Evidences in each subset will be fused with weights to get the aggregative center of *C_r_*, and the weighting fusion is based on information entropy.

Information entropy indicates the amount of information an evidence has. The larger the information entropy is, the less amount of information the evidence has. Define that the information entropy of evidence *M_l_* = (*m*_1_*_l_,m*_2_*_l_*,…,*m_nl_*) is calculated as follows [21]:

(23) $$H_{l}=\overset{n}{\underset{j=1}{\text{∑}}}m_{jl}\ln m_{jl}$$

If *Θ* is a focal element in *M_l_*, then a larger *m_l_*(Θ) means *M_l_* has less amount of information, so the amount of information of *M_l_* can be calculated according to the following formula:

(24) $$V_{l}=\left[1-m_{l}\left(\Theta \right)\right]\times f\left(H_{l}\right)=\left[1-m_{l}\left(\Theta \right)\right]\times e^{-H_{l}}$$

A smaller weight will be assigned to an evidence which has less amount of information, so the weight allocation of each evidence is as follows:

(25) $$W_{l}=\{\begin{array}{c} V_{l}/{\text{∑}}_{l=1}^{\left|C_{r}\right|}V_{l},if\exists l\text{where}V_{l}\neq 0\\ 1/\left|C_{r}\right|,\text{if}\forall l,V_{l}=0 \end{array}$$

Then we can get the aggregative center of *C_r_* by using an improved D-S formula. For any focal element “A”, the result of fusion is *n_r_*(*A*) = *p*(*A*) + *Kq*(*A*), where:

(26) $$p\left(A\right)=\underset{\cup_{i=1}^{\left|C_{r}\right|}A_{i}=A}{\text{∑}}\prod_{l=1}^{\left|C_{r}\right|}m_{l}(A_{i})$$

(27) $$q\left(A\right)=\overset{\left|C_{r}\right|}{\underset{l=1}{\text{∑}}}W_{l}\times m_{l}(A)$$

(28) K=Δ

Here, *p*(A) represents the traditional way to fuse evidences and *q*(A) represents the average support degrees from each evidence to *A*. When *K* is large enough, the influence from *q*(A) is increased. Assume that there are m subsets and *n_r_* is the aggregative center of *C_r_*, then the final result of fusion is:

(29) $$\text{m}\left(\text{A}\right)=\left({\text{∑}}_{k=1}^{m}\left(CW_{k}\times n_{k}(A)\right)\right)/\left(\underset{A}{\text{∑}}m(A)\right)$$

## 4. Simulation Analysis

### 4.1. Simulation Setting

**Figure 3.**
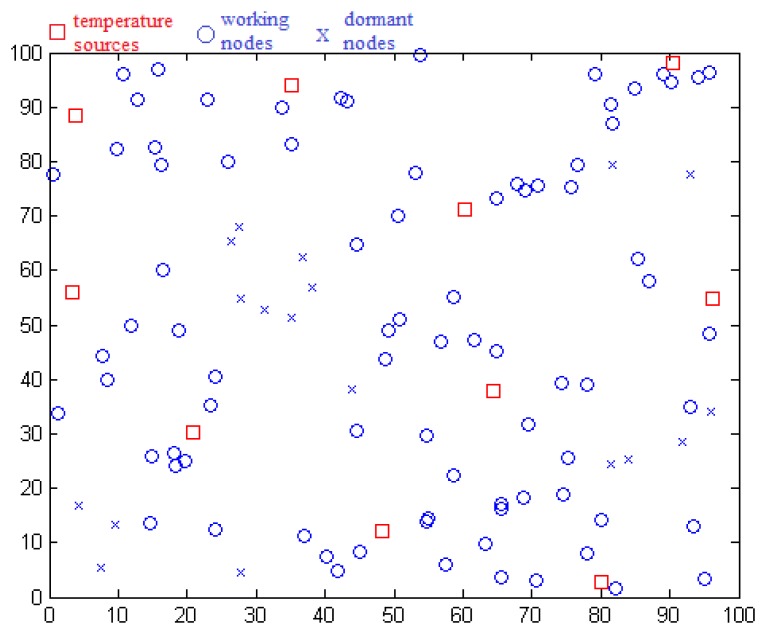
Topology description.

We use the MATLAB simulation tool to demonstrate our model. As shown in Figure 3, a square with a side length of 100 m is constructed in our model, in which sensors are deployed and form the network. Ten temperature sources are deployed in the square as the sensing objects of sensors. The distance between two sources is no less than *L*. Every temperature source randomly generates temperature data *x* which ranges from −5 to 40 °C. These readings simulate the temperature variation of four seasons, which means it has regularity and smoothness. Second, *n* sensors are deployed in this square and each of them selects the nearest temperature source which must be in the sensing range. If no temperature source exists within sensing range, a sensor is set to not work, which means no sensing from a temperature source and no communication with neighbor nodes.

Each working node establishes its variation of sensed data according to the distance to its temperature source, which can be described by the formulas below:

(30) $$X_{\max}=x+d/10$$

(31) $$X_{\min}=x-d/10$$

*X_max_* and *X_min_* are the upper and lower bounds of the data range, respectively. *x* is the temperature generated by the temperature sources and it is uniformly distributed in (*X_min_, X_max_*). *d* is the distance between a sensor and its temperature source. In every sensing moment, a sensor chooses a random value between *X_max_* and *X_min_* as its sensing data.

Each sensor node chooses other nodes which are within its communication range (communication radius is represented by *R*) and have the same temperature source as its neighbor nodes. After this, each node creates a set of neighbor nodes and the wireless sensor network is formed.

Two cases of uniformly distributed fault nodes and intensively distributed fault nodes are simulated. The first case is used in comparison when the number of nodes ranges. In the second case, we set squares which are located at a random coordinate as a fault region. We compare the detection effects for different scales of intensive faults by changing the area of a square. According to the sensing data designation, data ranging from *X_min_* to *X_max_* are treated as good, otherwise, data are treated as faulty. Fault data are set to *X_max_* + 5*d/r* or *X_min_* − 5*d/r*. Parameters are initialized: *L* = 30 m, *r* = 20 m, *R* = 20 m. In our simulation, each final data result is the average of results from 30 repeats.

### 4.2. Simulation Result Analysis

#### 4.2.1. Effect of Data loss

At each moment of the data collection, some nodes are chosen to be unable to sense data to simulate a data loss scenario. The Data Missing Preprocess Mechanism proposed in this paper is compared with the Data Filling method based on the *k*-Nearest Neighbor algorithm (df-KNN) algorithm. The main idea of df-KNN is to select *k* nodes from neighborhood which have the shortest distances, weigh the data of the *k* nodes according to these distances and finally sum the data as the interpolation result. Here, the data loss rate is set to 5%, 10%, 15%, 20%, 25%, 30% and 35%, respectively.

**Figure 4.**
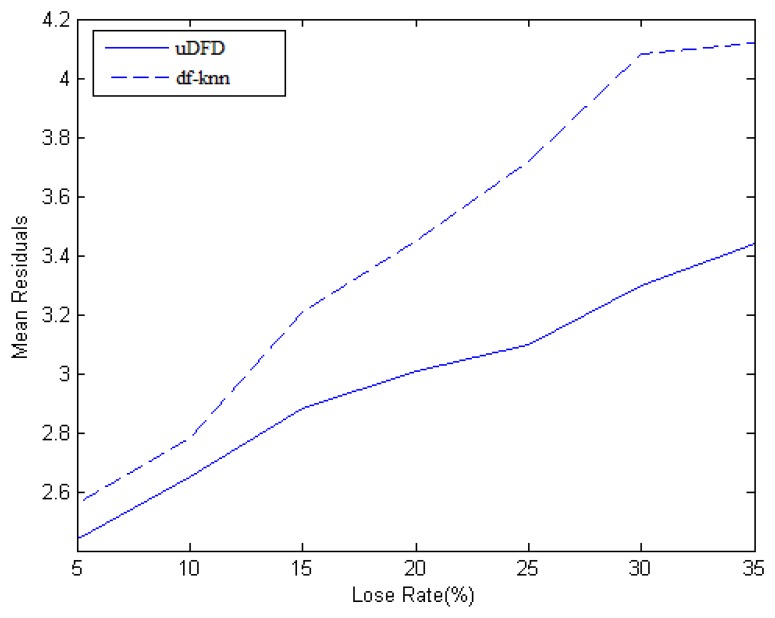
Effects of data filling.

As shown in Figure 4, data loss rate is set as the horizontal ordinate, which means the ratio of the number of the data loss nodes to the sum of working nodes. The mean residual is set as the vertical ordinate, which means the average of differences between interpolation data and pre-established data and it reflects the final accuracy of the algorithms. Mean residual grows as the loss rate grows. When the loss rate is lower, the mean residual of uDFD is 0.1 lower than that of KNN, and achieves an unremarkable improvement, but as the loss rate grows higher, the improvement turns to be higher. Approximately, when the loss rate is high enough, the improvement is 0.5, which means uDFD is more suitable in the large-scale data loss situation. With the growth of data loss rate, the number of neighbors which have available data reduces, which means less information could be collected and eventually this makes the interpolation results unreliable. In comparison with df-KNN, uDFD adequately involves the historical data of neighbors to predict and solve the problem of credit reduction due to less available data, which leads to better results.

#### 4.2.2. Evidence Fusion

In this paper, information theory-based evidence reasoning is used to fuse collected evidences before the status judgment of nodes. Original D-S evidence reasoning and an improved one proposed by Qiang Ma *et al.* [22] are used for comparison. The improved D-S is depicted as below.

Define the distance between evidence *m_1_* and *m_2_*:

(32) $$\text{d}\left(m_{1},m_{2}\right)=\sqrt{1/2{\left({\text{m}}_{1}-m_{2}\right)}^{T}(m_{1}-m_{2})}$$

Define the similarity of *m_1_* and *m_2_*:

(33) $$\text{S}\left(m_{1},m_{2}\right)=1-\text{d}(m_{1},m_{2})$$

Define the basic credit of *m_i_*:

(34) $$\beta_{i}={\text{∑}}_{1\leq j\leq N,j\neq i}s(m_{1},m_{2})$$

Here *N* is the sum of evidences.

Define the weight of *m_j_*:

(35) $$\phi_{i}=\beta_{i}/\underset{1\leq j\leq N}{\max}\beta_{j}$$

Amend all evidences:

(36) $$m_{i}^{\prime }\left(A\right)=\varphi_{i}m_{i}(A)$$

(37) $$m_{i}^{\prime }\left(\psi \right)=\varphi_{i}m_{i}\left(\psi \right)+(1-\varphi_{i})$$

*A* is the established focal element and *ψ* is the uncertain one. Fuse the amended evidences through the original D-S. The algorithm above measures the degree of conflicts among evidences by involving distance and amends evidences before fusion. The simulation results are shown in Table 1.

Belief function and plausibility function are involved to estimate the fusion results. BPA-based belief function in the frame of discernment Θ is defined as:

(38) $$\text{Bel}\left(\text{A}\right)=\underset{B\subseteq A}{\text{∑}}m(B)$$

BPA-based plausibility function in the frame of discernment Θ is defined as:

(39) $$\text{Pl}\left(\text{A}\right)=\underset{B\cap A\neq \emptyset }{\text{∑}}m(B)$$

**Figure 5.**
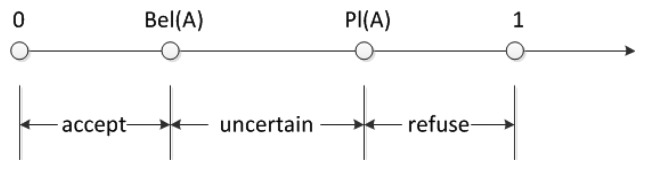
Belief interval.

Belief interval is defined as [*Bel*(*A*), *Pl*(*A*)], which is shown in Figure 5. The hypothesis that *A* is true is accepted in [0, *Bel*(*A*), is uncertain in [*Bel*(*A*), *Pl*(*A*)] and is refused in *Pl*(*A*), 1]. The length of interval presents the possibility to make a corresponding conclusion to this hypothesis.

The belief functions and plausibility functions are shown in Table 2 according to the fusion results.

The results of these algorithms are similar to each other when two evidences with low degree of conflict in evidence set 1 are to be fused, among which the results of the original D-S and the improved D-S proposed by Qiang Ma [22] are the same. A similar evidence is added to set 1 to form set 2. Through the analysis of belief functions and plausibility functions of three algorithms, the possibility of original D-S to accept that *G* is true is 0.2703 and the possibility to refuse is 0.3514. The possibility of improved D-S to accept that *G* is true is 0.2561 and the possibility to refuse is 0.3388. The possibility of uDFD to accept that *G* is true is 0.1956 and the possibility to refuse is 0.2272. According to evidence set 2, the possibilities of the previous two algorithms to accept and refuse that *G* is true is too high to reflect the actual situation (the possibilities to accept and refuse are both lower than 0.2) of each evidence. However, the algorithm proposed in this paper is closer. In uDFD, the possibilities to accept and refuse are not raised by reducing the uncertainty, which makes the fusion result more credible. A completely different evidence is added to set 1 to form set 3 with high degree of conflict. It is obvious that the possibility (0.5978) of original D-S to accept that *G* is true is so high that approaches the one of the last evidence which is added in set 3 and the possibility (0.2881) of improved D-S is too low to reflect the affect caused by high degree of conflict. The result of uDFD is between the ones of the previous two algorithms, which balances the influences of all evidences and is more credible.

#### 4.2.3. Detection Accuracy

DFD, IDFD and uDFD are compared based on the constructed wireless sensor network model. Measures to be involved are detection accuracy (the ratio of number of correctly detected nodes to the sum of working nodes), false alarm rate (the ratio of number of nodes which are misjudged from good to false to the sum of working nodes), missing alarm rate (the ratio of number of nodes which are misjudged from false to good to the sum of working nodes). In this simulation, each final data point is the average of results from 30 repeats.

First, we analyze the effects of these three algorithms with the changing fault rate when nodes are randomly distributed uniformly. Considering that different influences are caused by different distribution densities, cases with 40, 80 and 120 working nodes are simulated.

**Figure 6.**
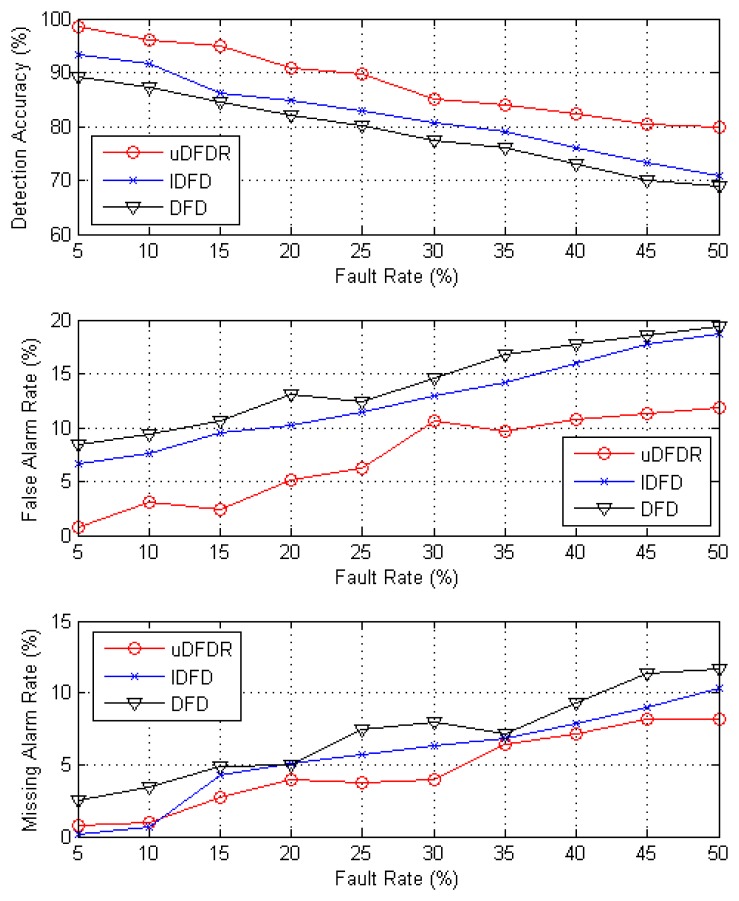
Detection effects under 40 working node conditions.

Figure 6 shows the detection effects of the three algorithms with 40 working nodes. In this case, nodes are distributed sparsely in the simulation region. It can be seen from the figure that with the increasing fault rate, detection accuracy shows an approximate linear downward trend; however, false alarm rate and missing alarm rate show the opposite trend. Through further calculation, when the fault rate ranges from 5% to 50%, average detection accuracy of uDFD is 9.33% points higher than that of DFD and 6.25% points higher than that of IDFD; average fault alarm rate of uDFD is 6.93% points lower than that of DFD and 5.33% points lower than that of IDFD; average missing alarm rate of uDFD is 2.49% points lower than that of DFD and 1.02% points lower than that of IDFD. The uDFD brings better detection accuracy under sparse distribution conditions.

**Figure 7.**
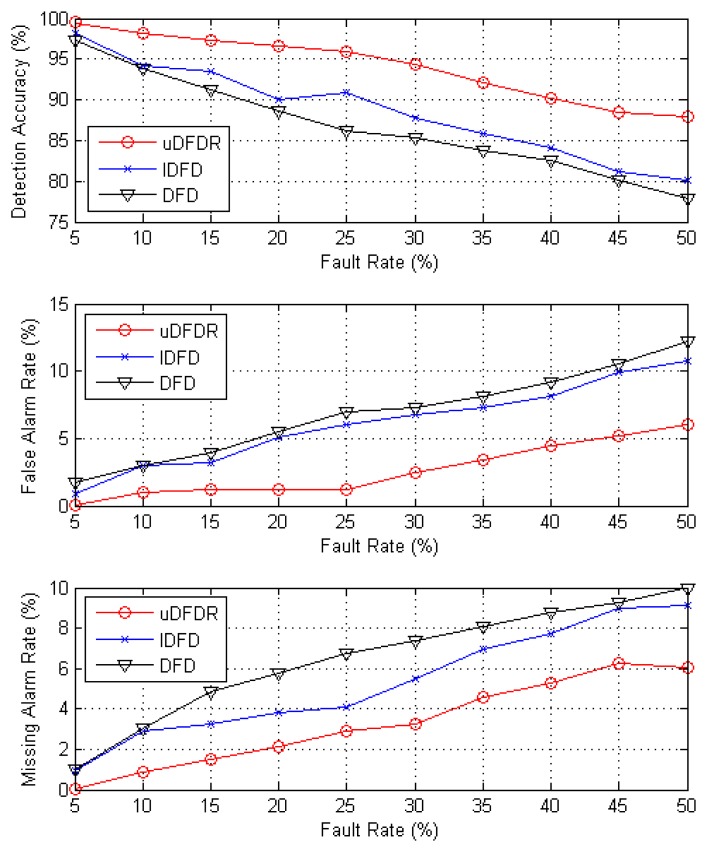
Detection effects under 80 working node conditions.

Figure 7 shows the detection effects of the three algorithms with 80 working nodes. In this case, nodes are distributed moderately densely in the simulation region. As is shown by the figure, with the increasing fault rate, detection accuracy shows an approximately linear downward trend; however, false alarm rate and missing alarm rate show the opposite trend. Through further calculation, when the fault rate ranges from 5% to 50%, average detection accuracy of uDFD is 7.31% points higher than that of DFD and 5.44% points higher than that of IDFD; average fault alarm rate of uDFD is 4.21% points lower than that of DFD and 3.4% points lower than that of IDFD; average missing alarm rate of uDFD is 3.22% points lower than that of DFD and 2.04% points lower than that of IDFD. The uDFD provides better detection accuracy in this condition. Meanwhile, as the fault rate grows, the superiority of the detection effect of uDFD continues to increase. When the fault rate is 50%, the detection accuracy of uDFD is 10.08% points higher than that of DFD and 7.68% points higher than that of IDFD, which indicates that uDFD adapts better to high fault rate conditions.

**Figure 8.**
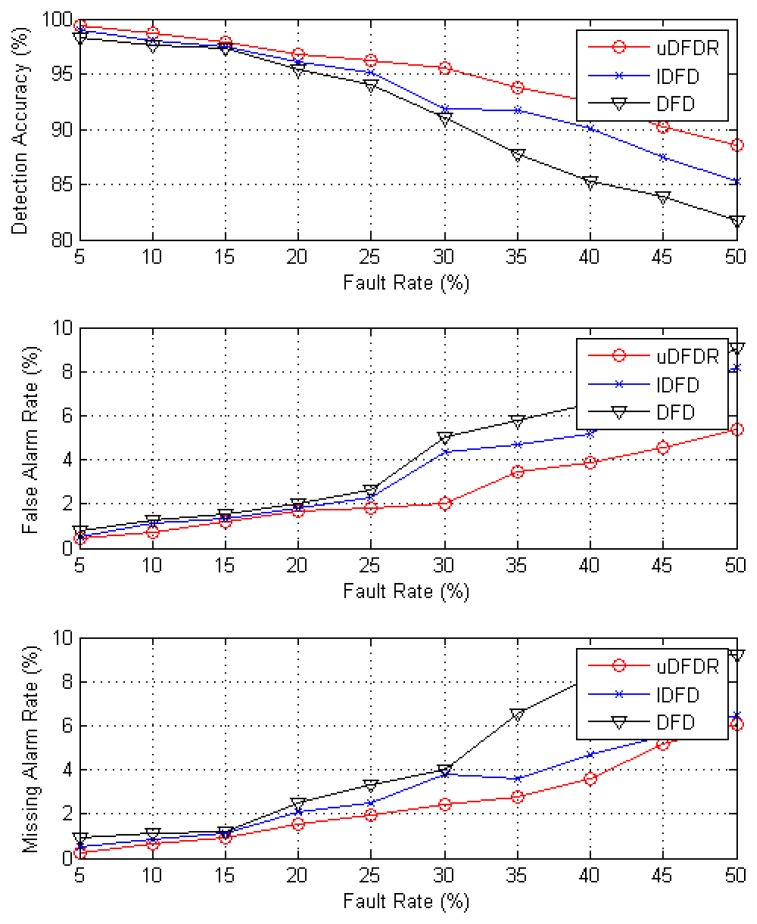
Detection effects under 120 working node conditions.

Figure 8 shows the detection effects of the three algorithms with 120 working nodes. In this case, nodes are distributed densely in the simulation region. It can be seen from the figure that with the increasing fault rate, detection accuracy shows an approximately linear downward trend; however, false alarm rate and missing alarm rate show the opposite trend. Through further calculation, when the fault rate ranges from 5% to 50%, the average detection accuracy of uDFD is 3.75% points higher than that of DFD and 1.74% points higher than that of IDFD; average fault alarm rate of uDFD is 1.69% points lower than that of DFD and 1.14% points lower than that of IDFD; average missing alarm rate of uDFD is 2.09% points lower than that of DFD and 0.6% points lower than that of IDFD. The uDFD provides better detection accuracy under dense distribution conditions. Furthermore, as the fault rate grows, the superiority of the detection effect of uDFD increases continually, which indicates that uDFD performs better under high fault rate conditions.

Through comprehensive analysis of Figures 6, 7 and 8, we can see that detection accuracy of these algorithms increases with the increasing distribution density of nodes. This is due to the increase of available information when judging resulting from the growing number of neighbor nodes. By comparison, the advantage of detection accuracy of uDFD increased as the distribution density of nodes decreases, which indicates that the detection accuracy of uDFD improves more than that of DFD and IDFD. Thanks to evidence fusion based on the status of neighbor nodes before judgment, uDFD works better under small number of neighbor node conditions.

Second, we analyze the detection accuracy of the three algorithms when fault nodes are intensively distributed. The intensive distribution scheme involves setting squares located at random coordinates with length of 20, 25, 30, 35 and 40 m as the fault regions. In the fault region, all nodes are set to fault. By changing the size of the ault region, we can observe the detection accuracy for different scales of faulty nodes. Here, the number of working nodes is 80.

**Figure 9.**
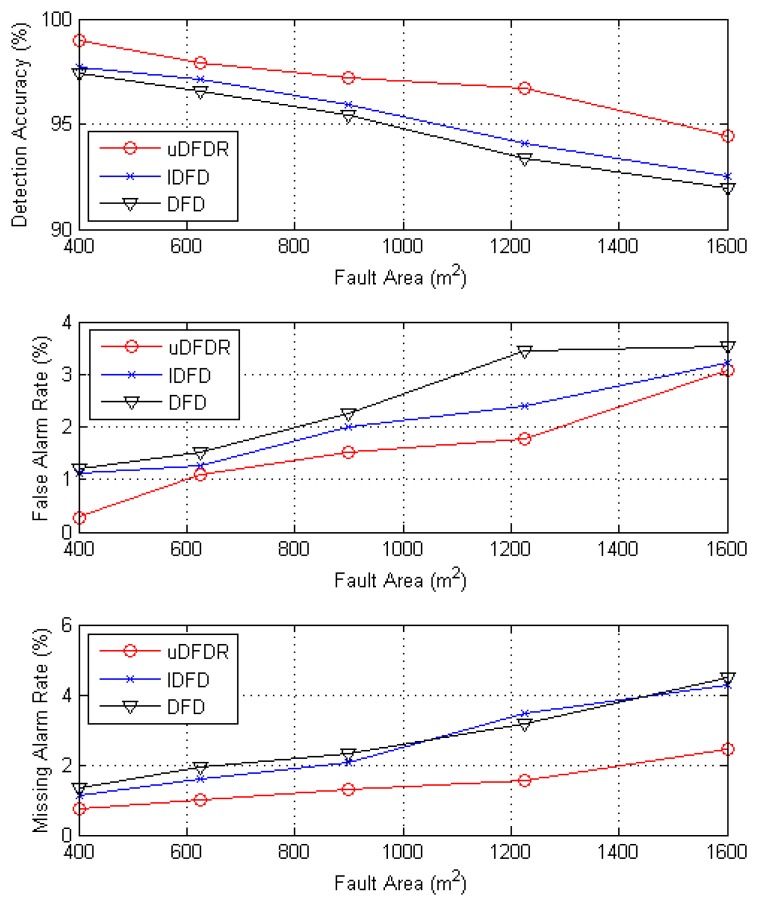
Detection effects under intensive fault conditions.

Figure 9 shows detection effects of the three algorithms with different fault region sizes. When the fault rate ranges from 5% to 50%, the average detection accuracy of uDFD is 2.08% points higher than that of DFD and 1.55% points higher than that of IDFD; average fault alarm rate of uDFD is 0.84% points lower than that of DFD and 0.45% points lower than that of IDFD; average missing alarm rate of uDFD is 1.24% points lower than that of DFD and 1.09% points lower than that of IDFD. It is easy to conclude that uDFD can achieve better detection effects when intensive faults occur. The uDFD takes in more information from the neighborhood to judge when dealing with intensive faults situations, which reduces the influence from mutual cheating among faulty nodes.

In uDFD, *θ_1_* is the threshold of data from different nodes on same moment, *θ_2_* is the threshold of data from the same node on different moments and *θ_3_* is the threshold of data of the same node collected on the same moment of different days. In our model, a value of *θ_2_* ranging within the interval (0, 2) has little influence on detection effect, so it is set to a fixed value of 1. Based on those values, the best combination of *θ_1_* and *θ_3_* is going to be explored.

**Figure 10.**
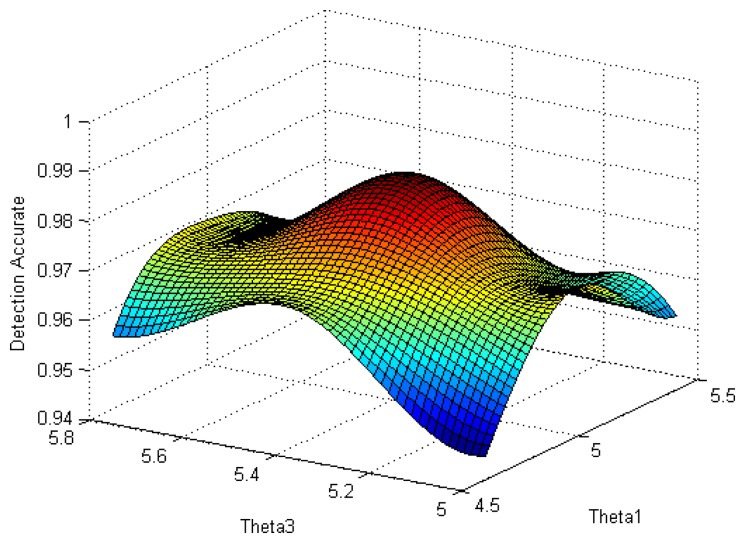
Detection accuracy with different combinations of *θ_1_* and *θ_3_*.

The selection of *θ_1_* and *θ_3_* is directly related to the judgment results, thus, to explore the best combination of *θ_1_* and *θ_3_* becomes the key to explore the best detection effect of uDFD. Figure 10 shows a 3D map of detection accuracy with different combinations of *θ_1_* and *θ_3_*. Here, the number of working nodes is 80. It can be seen from the curved surface that the combination of *θ_1_* = 5, *θ_3_* = 5.4 approaches the peak, which means the maximum detection accuracy is 0.98.

#### 4.2.4. Communication Energy Consumption

**Figure 11.**
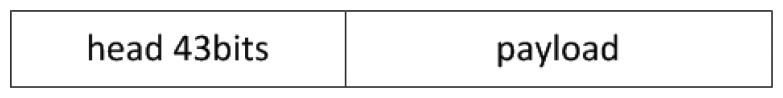
Brief frame structure of ZigBee.

In our model, communication between nodes is simulated by using the ZigBee protocol. ZigBee, a personal area network protocol based on IEEE802.15.4, supports short-distance, low-complexity, self-organizing, low-power, high-speed and low-cost wireless communication technology and applies well in WSNs. The brief frame structure of ZigBee is shown in Figure 11. The frame head is constructed by the bits from the application layer, network layer, MAC layer and physical layer. In our model, messages transmitted between nodes include prejudged statuses, evidences and final judged statuses. The information above can be encapsulated in the payload field of the application layer frame by analyzing the frame structure of ZigBee. When statuses are to be transmitted, the payload is only 4 bits (2 bits present message type and 2 bits present status, namely *LG/G*) and the total length of a frame is 47 bits (the header length is 43 bits).

Radio energy dissipation model is calculated as indicated below:

(40) $$\begin{array}{ll} E_{Tx}(l,d) & =E_{Tx-\text{elec}}(l)+E_{Tx-\text{amp}}(l,d)\\ & =\{\begin{array}{c} lE_{\text{elec}}+l\epsilon_{fs}d^{2},d<d_{0}\\ lE_{\text{elec}}+l\epsilon_{mp}d^{4},d\geq d_{0} \end{array} \end{array}$$

(41) $$E_{Rx}\left(l\right)=E_{Rx-\text{elec}}\left(l\right)=lE_{\text{elec}}$$

*E_Tx_*(*l, d*) is the transmission energy, which includes electronic energy *E_Tx_*_−_*_elec_*(*l*) and amplifier energy *E_Tx_*_−_*_amp_*(*l, d*). *l* is the length of a message. *E_elec_* is determined by digital coding, modulation and filtering is fixed as 50 nJ/bit. *ϵ_fs_* = 10 pJ/bit/m^2^ and *ϵ_mp_* = 0.0013 pJ/bit/m^4^. *d* is the transmission distance. *d_0_* is the threshold of *d* and is set to 20 m. If *d* is larger than *d_0_*, energy dissipation is mainly caused by free space power loss (*d*^2^). Otherwise, energy dissipation is mainly caused by multipath power loss (*d*^4^).

**Figure 12.**
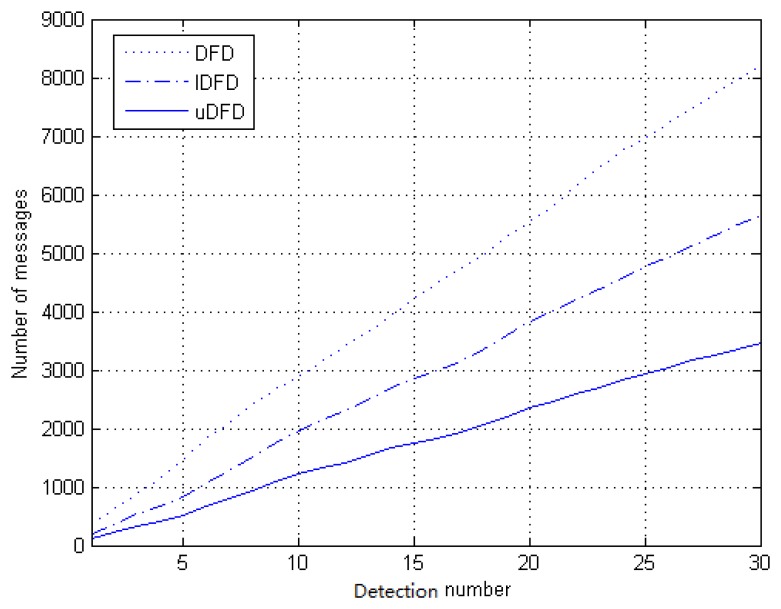
Number of messages accumulated during 30 tests under 40 working node conditions.

First, we analyze the number of messages transmitted in the simulated network under 40 working node conditions, which is depicted in Figure 12. When transmitting messages, nodes exchange statuses by radio broadcasting. An accumulated number of messages is recorded in the process of 30 tests. With the growth of test rounds, the number of messages shows an approximately linear upward trend. Through further calculation, approximate slopes of DFD, IDFD and uDFD are 266.7, 180.0 and 103.3, respectively. Obviously, the rate of increase of uDFD is the lowest, which means a minimum of messages transmitted during the fault detection. This is because the nodes in DFD and IDFD have to exchange all prejudged states and final judged statuses whether they are good or faulty, which leads to more interactions, while uDFD only exchanges message if the tendency status is *LG* or final status is good.

**Figure 13.**
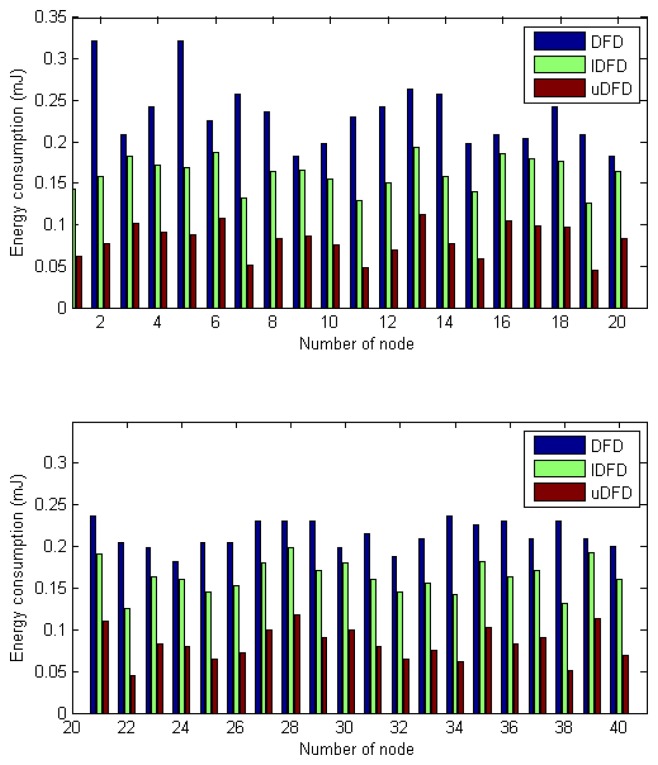
Energy consumption of each node under 40 working node conditions.

The comparison of average communication energy consumption of each node after 30 tests is shown in Figure 13. Average energy consumptions of all nodes in DFD, IDFD and uDFD are 0.235, 0.176 and 0.082 mJ. By counting and comparing the energy consumption of each node specifically, we find that uDFD has the best energy saving performance, which is caused by the reduction of interactions.

**Figure 14.**
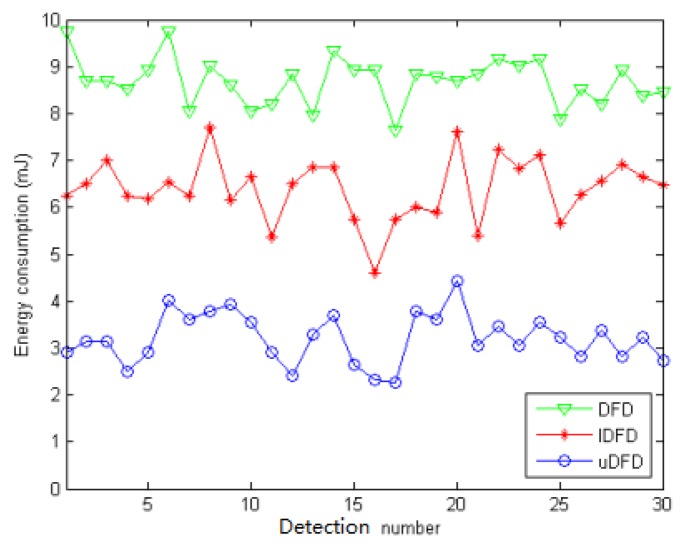
Energy consumption of each detection under 40 working node conditions.

**Figure 15.**
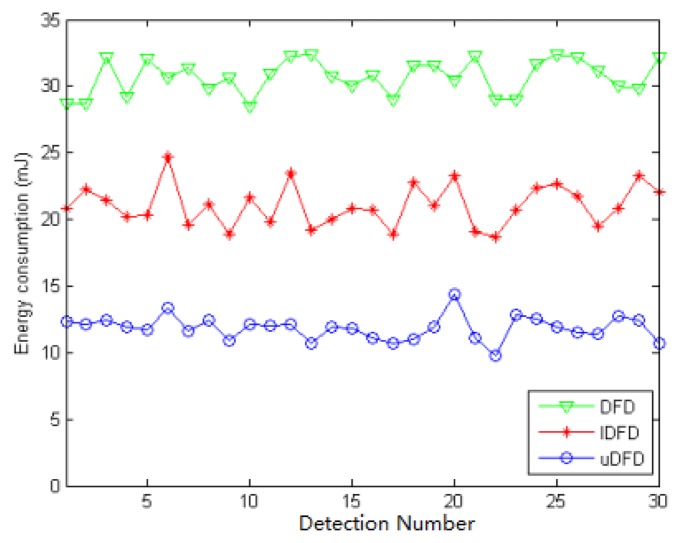
Energy consumption of each detection under 80 working node conditions.

**Figure 16.**
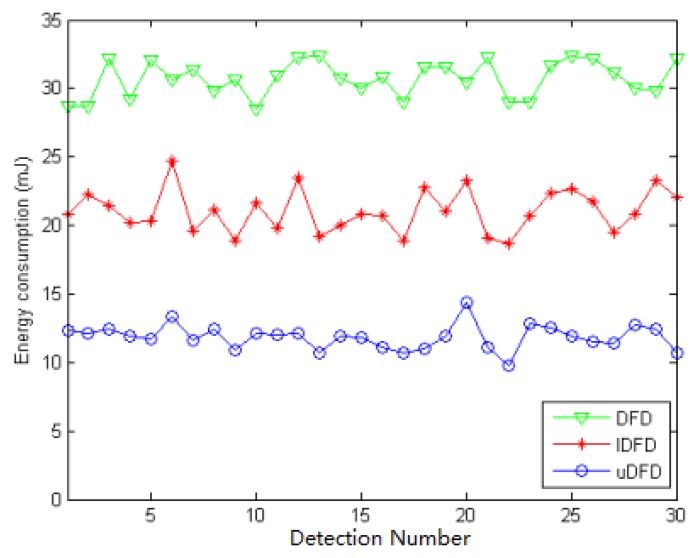
Energy consumption of each detection under 120 working node conditions.

Figures 14, 15 and 16 show the energy consumption of each detection under conditions of 40, 80 and 120 working nodes. Through further calculation, under 40 working node conditions, the average energy consumption of all detections in uDFD is 2.87 mJ lower than that of IDFD and 5.47 mJ lower than that of DFD. Under 80 working node conditions, the average energy consumption of all detections in uDFD is 4.77 mJ lower than that of IDFD and 10.6 mJ lower than that of DFD.

Under 120 working node conditions the average energy consumption of all detections in uDFD is 9.26 mJ lower than that of IDFD and 19.59 mJ lower than that of DFD. The uDFD has the best energy saving performance during detection. Compared with DFD and IDFD, uDFD has less iteration and no need to transmit *Un* status, which reduces interactions and decreases energy consumption. Messages carrying evidences use more bits than those carrying status, but this disadvantage has little adverse influence on the overall performance of uDFD.

We achieve good performances which are shown in simulation above. As traditional DFD and IDFD require that each node broadcast its status, we reduce the communication overload by broadcasting the status of nodes which are determined as good. What's more, uDFD displays higher detection accuracy in a high data loss rate environment.

## 5. Conclusions

In the paper, we propose a fault detection mechanism for wireless sensor networks based on data filling and evidence fusion methods. Aiming at decreasing of detection accuracy due to data losses, the uDFD mechanism is demonstrated to be more suitable in the large-scale data loss situation. What's more, information entropy theory-based evidence reasoning is used to fuse collected evidences before the status judgment of nodes. This helps balance the influences of all evidences and make them more credible. Our algorithm can retain higher detection accuracy regardless of lower connectivity environment or changing fault ratios. The design that only sensors determined as good require exchanging states for evidence fusion decreases the number of messages broadcast in the process of fault detection. Avoiding too many message exchanges for fault detection will reduce a huge burden on the limited energy of sensors. In the future, we will solve the phenomenon that the detection accuracy is less than 80% when the fault ratio is closer to 0.5. For example, we will consider historical judgment behaviors to reason and increase detection accuracy, as well as the cross-impact of more types of faults.

## Figures and Tables

**Table 1.** Results of evidence fusion.

**Evidences**	**Evidence Set 1 m_1_:m(G) = 0.1, m(F) = 0.2, m(ψ) = 0.7 m_2_:m(G) = 0.2, m(F) = 0.2, m(ψ) = 0.6**	**Evidence Set 2 m_1_:m(G) = 0.1, m(F) = 0.2, m(ψ) = 0.7 m_2_:m(G) = 0.2, m(F) = 0.2, m(ψ) = 0.6 m_3_:m(G) = 0.1, m(F) = 0.1, m(ψ) = 0.8**	**Evidence Set 2 m_1_:m(G) = 0.1, m(F) = 0.2, m(ψ) = 0.7 m_2_:m(G) = 0.2, m(F) = 0.2, m(ψ) = 0.6 m_3_:m(G) = 0.6, m(F) = 0.2, m(ψ) = 0.2**

**Algorithms**			
Orginal D-S	**m(G) = 0.234,**	**m(G) = 0.2703,**	**m(G) = 0.5978,**
	**m(F) = 0.3191,**	**m(F) = 0.3514,**	**m(F) = 0.2849,**
	**m(ψ) = 0.4468**	**m(ψ) = 0.3784**	**m(ψ) = 0.1173**

Improved D-S by Qiang Ma	**m(G) = 0.234,**	**m(G) = 0.2561,**	**m(G) = 0.2881,**
	**m(F) = 0.3191,**	**m(F) = 0.3388,**	**m(F) = 0.2909,**
	**m(ψ) = 0.4468**	**m(ψ) = 0.2272**	**m(ψ) = 0.4210**

uDFD	**m(G) = 0.2271,**	**m(G) = 0.1956,**	**m(G) = 0.3384,**
	**m(F) = 0.3088,**	**m(F) = 0.2272,**	**m(F) = 0.2679,**
	**m(ψ) = 0.4641**	**m(ψ) = 0.5772**	**m(ψ) = 0.3972**

**Table 2.** Belief functions and plausibility functions of fusion results.

	**Evidence Set 1**	**Evidence Set 2**	**Evidence Set 3**
Original D-S	**Bel(G) = 0.234, Pl(G) = 6808**	**Bel(G) = 0.2703, Pl(G) = 0.6486**	**Bel(G) = 0.5978, Pl(G) = 0.7151**
	**Bel(F) = 0.3191, Pl(F) = 0.7659**	**Bel(F) = 0.3514, Pl(F) = 0.7291**	**Bel(F) = 0.2849, Pl(F) = 0.4022**
	**Bel(ψ) = 0.4468, Pl(ψ) = 1**	**Bel(ψ) = 0.3784, Pl(ψ) = 1**	**Bel(ψ) = 0.1173, Pl(ψ) = 1**

Improved D-S by Qiang Ma	**Bel(G) = 0.234, Pl(G) = 6808**	**Bel(G) = 0.2561, Pl(G) = 0.6612**	**Bel(G) = 0.2881, Pl(G) = 0.7091**
	**Bel(F) = 0.3191, Pl(F) = 0.7659**	**Bel(F) = 0.3388, Pl(F) = 0.7439**	**Bel(F) = 0.2909, Pl(F) = 0.7119**
	**Bel(ψ) = 0.4468, Pl(ψ) = 1**	**Bel(ψ) = 0.4051, Pl(ψ) = 1**	**Bel(ψ) = 0.4210, Pl(ψ) = 1**

uDFD	**Bel(G) = 0.2271, Pl(G) = 0.6912**	**Bel(G) = 0.1956, Pl(G) = 0.7728**	**Bel(G) = 0.3348, Pl(G) = 0.7321**
	**Bel(F) = 0.3088, Pl(F) = 0.7729**	**Bel(F) = 0.2272, Pl(F) = 0.8044**	**Bel(F) = 0.2679, Pl(F) = 0.6652**
	**Bel(ψ) = 0.4641, Pl(ψ) = 1**	**Bel(ψ) = 0.3784, Pl(ψ) = 1**	**Bel(ψ) = 0.3972, Pl(ψ) = 1**
